# Numerical Simulation and Analysis of Turbulent Characteristics near Wake Area of Vacuum Tube EMU

**DOI:** 10.3390/s23052461

**Published:** 2023-02-23

**Authors:** Hongjiang Cui, Guanxin Chen, Ying Guan, Huimin Zhao

**Affiliations:** 1 School of Locomotive and Rolling Stock Engineering, Dalian Jiaotong University, Dalian 116028, China; 2 School of Electronic Information and Automation, Civil Aviation University of China, Tianjin 300300, China; 3 Traction Power State Key Laboratory of Southwest Jiaotong University, Chengdu 610031, China

**Keywords:** vacuum tube, EMU, wake, turbulence characteristics, CFD

## Abstract

Due to aerodynamic resistance, aerodynamic noise, and other problems, the further development of traditional high-speed electric multiple units (EMUs) on the open line has been seriously restricted, and the construction of a vacuum pipeline high-speed train system has become a new solution. In this paper, the Improved Detached Eddy Simulation (IDDES) is used to analyze the turbulent characteristics of the near wake region of EMU in vacuum pipes, so as to establish the important relationship between the turbulent boundary layer, wake, and aerodynamic drag energy consumption. The results show that there is a strong vortex in the wake near the tail, which is concentrated at the lower end of the nose near the ground and falls off from the tail. In the process of downstream propagation, it shows symmetrical distribution and develops laterally on both sides. The vortex structure far from the tail car is increasing gradually, but the strength of the vortex is decreasing gradually from the speed characterization. This study can provide guidance for the aerodynamic shape optimization design of the rear of the vacuum EMU train in the future and provide certain reference significance for improving the comfort of passengers and saving the energy consumption caused by the speed increase and length of the train.

## 1. Introduction

As a symbol of the development of railway transportation, increasing the speed of rail trains has always been a goal, an objective demand, and a dream that related researchers have been pursuing [1,2,3]. However, an increase in speed inevitably brings many problems that can be ignored at low speeds, such as aerodynamic drag, aerodynamic noise, and aerothermal effect [4,5,6,7]. At present, hundreds of millions of tons of oil are consumed in transportation on the earth every year, which wastes resources and damages the environment [8,9,10]. Building a high-speed transportation network with low energy consumption and environmental protection has become a very urgent need. China’s high-speed rail development is now at the forefront of the world. It is necessary to look farther to build an ultra-high-speed vacuum pipeline train network with a speed of more than 1000 km per hour [11,12,13,14].

A series of aerodynamic problems, such as train passing, crosswind effect, tunnel effect, aerodynamic drag, and wake, etc. will occur when trains are running at high speeds in the open air. Many scholars have conducted in-depth studies on these issues. Baker [15,16] drew lessons from a wide range of train model size and full-scale experiments and calculations, trying to establish a comprehensive flow field diagram. Additionally, for still air conditions and crosswind conditions, the researchers are limited to the train in the open air. Hemida et al. [17] analyzed the flow in the near-wake region under transient conditions and believed that the three-dimensional turbulence in the near-wake region has a complex turbulence structure. Choi et al. [18] verified the feasibility of dynamic control (active control or adaptive control) in the numerical simulation of channel turbulence. Huang et al. [19] compared the four algorithms and obtained the setting method for the calculation of the external flow field of the vacuum pipeline train, which provides a theoretical basis for the simulation calculation. Meanwhile, the characteristics of the distribution of turbulent kinetic energy in the wake area and the correlation between train wind and vortex shedding were discussed. Pan [20] applied the flat-plate boundary layer theory to a detailed comparative analysis of the thickness of the surface boundary layer of the high-speed train body and the distribution characteristics of the surface friction resistance, deriving the three-dimensional average motion equation. Meanwhile, the characteristics of the distribution of turbulent kinetic energy in the wake area and the correlation between train wind and vortex shedding were discussed. Bell et al. [21,22,23,24] found periodic vortex shedding in the near wake region of high-speed trains in the 1:10 wind tunnel test and discussed the flow changes of the topological structure of wake vortices and the relationship between train wind and wake vortex structure. Some researchers use the Proper Orthogonal Decomposition (POD) method to study the flow characteristics of the vortex in the wake of a high-speed train. Liu et al. [25] studied the dynamic characteristics of a high-speed train wake vortex in the speed range of 200~450 km/h and analyzed the order reduction of strong unsteady flow in the train wake region. Muld et al. [26,27,28] extracted the flow field structure near the wake region of the train model, identified a pair of counter-rotating flow vortices, obtained three dominant frequencies of vortex shedding, and found the bending motion of vortex pairs in the evolution process. Xia et al. [29,30,31] clarified the dynamic characteristics of the near wake. In addition, the IDDES method is used to study the effects of ground structure and ground effect on the slip flow and near wake of a high-speed train. Wang [32] studied the vortex structure in the wake region of a high-speed train under different Reynolds numbers. With the increase of the Reynolds number, some small-scale vortices appeared in the wake region of a high-speed train, but the large-scale vortex structure did not change significantly. In Yao’s research on the wake characteristics of high-speed trains, it was concluded that the optimization of the aerodynamic performance of the tail car should aim at reducing the strength of the tail vortex system [33]. Sterling et al. [34] obtained some conclusions on the characteristics of train slipstreams by comparing all available data sets of high-speed passenger trains and container freight trains. Kim et al. [35] selected a high-speed train in actual operation and a tunnel in the service section and evaluated the pressure characteristics of a single train through numerical analysis and experiments. Shin et al. [36] used the three-dimensional unsteady Navier-Stokes equation solver to explore the changes in aerodynamic force and the generation of compression waves when high-speed trains enter the tunnel. Gallani et al. [37] studied the aerodynamic performance of vacuum pipeline trains. The result shows that under appropriate vacuum pressure, different shapes of train heads and tails have a significant impact on the drag force of vacuum trains in the tunnel. Zhong et al. [38] used an improved delayed separation eddy simulation (IDDES) method to study two typical flow fields under different blockage rates and found that as the vacuum degree increases, the thickness of the wake boundary layer and the width of the vortex group increase slightly. Liang et al. [39] set the ballast height to 1.825 m to obtain the lowest slipstream velocity in the wake area of the high-speed train, which greatly limits the outward and downward movement of the vortex and improves the flow structure in the wake area. Li et al. [40] took the ICE-2 train model as the research object and studied the relationship between aerodynamic resistance and flow structure caused by train operation in time average and time-dependent views. Jia et al. [41,42] studied how train length affects the boundary layer, wake, surface pressure, aerodynamic resistance, and friction resistance. Zhou et al. [43,44,45] studied the fluctuation phenomenon produced by a vacuum pipeline maglev train at ultra-high speed, deduced the relationship between the critical blocking ratio and the critical incoming Mach Number in the vacuum pipe, and revealed the distribution characteristics of the flow field in the pipe. Kwon et al. [46,47] used the numerical calculation method to explore the basic characteristics of the flow field around the vacuum pipe train. Tan et al. [48] conducted a transient numerical simulation of maglev trains with different marshaling lengths under the condition of no wind in the open air and analyzed the characteristics of the wake structure. Sui et al. [49] studied the effect of vacuum on the flow field around the train cabin in a circular section vacuum tube. Dong et al. [50] studied the influence of ground clearance on simplified high-speed train flow, proposing and describing different topologies of train wake. Tian et al. [51,52] found that the eddy current around the train is mainly caused by the structure with complex mutation and large curvature change on the train surface, and put forward a series of drag reduction measures. In addition, some other methods have also been proposed in recent years [53,54,55,56,57,58,59,60,61,62,63,64,65,66,67,68,69,70,71,72].

At present, there is no three-dimensional numerical simulation of the turbulence characteristics in the wake of high-speed trains with vacuum ducts. Based on the research and analysis of the turbulence characteristics of the high-speed electric multiple unit (EMU) under the open line condition in the paper, a three-dimensional compressible model is used to numerically calculate the turbulent kinetic energy in the wake region of high-speed trains in the vacuum pipeline. Under the pressure of 0.01 atm and the speed of 800 km/h of a train in the vacuum pipeline, different train formation forms are used as variables to analyze the turbulence characteristics of the rear and wake regions of the EMU.

## 2. Numerical Simulation Method

### 2.1. Fluid Model

In the vacuum tube, when the temperature is constant, the gas density changes with the pressure in the pipe. As the pressure decreases and the density decreases, the gas thinning effect in the tube will become more obvious. A dimensionless parameter that characterizes the rarefied degree of gas in fluid mechanics is abbreviated as:

(1) Kn=λ/L

where *Kn* represents the Knudsen Number, *λ* represents the mean free path of air molecules, and *L* represents the flow characteristic size.

It is equal to the ratio of the mean free path of gas molecules to the characteristic length of the flow field (such as the linear scale of a spacecraft or satellite or the diameter of aerosol particles), and the larger the Knudsen Number, the thinner the gas. Therefore, the flow can be divided into the following types [73,74,75]: when the Knudsen Number is less than 0.01, the gas flow belongs to the category of continuous media. When the Knudsen Number is between 0.01 and 0.1, the Navier–Stokes equation with slip boundary conditions can be used to describe the fluid, which is called slip flow. When it is between 0.1 and 10, it belongs to the transition zone; when the Knudsen Number is greater than 10, the molecular assumption is adopted, and the Boltzmann equation is used to describe the fluid directly [76].

For gas molecules, the average distance between two adjacent collisions is called the mean free path of the molecule, and its expression is:

(2) $$\lambda =\frac{k_{B}T}{\sqrt{2}\pi d^{2}P}$$

where *P* represents the pressure in the pipeline, *k_B_* represents Boltzmann’s constant, which is 1.3805 × 10^−23^, *d* represents molecular effective diameter, and *T* represents the temperature in the pipeline.

When the temperature is 298 K and the pressure is 104 Pa, the mean free path of a molecule with an effective diameter of 3 × 10^−10^ is 1.028 × 10^−6^. For the flow around the high-speed train in the vacuum pipeline, the characteristic length of the flow can be taken as the minimum dimension of the space, that is, the distance between the high-speed train and the ground, which is set to 0.2 m [77,78,79]. According to formula 2, the corresponding value is 5.14 × 10^−6^, which is less than 0.01, belonging to the continuum model.

### 2.2. High-Speed Train Model and Calculation Domain

In this paper, a certain type of high-speed EMU is used as a prototype to establish a simulation model. The real EMU is usually composed of 8 cars with a total length of about 200 m, including pantographs, bogies, and other structures. If the numerical simulation calculation is performed on the whole vehicle, the calculation amount will be too large to achieve. Therefore, it is necessary to appropriately simplify the EMU model.

(1) Three length models (denoted as atm1, atm2, atm3) are adopted: head-1 carriage-tail train, head-2 carriages-tail train and head-3 carriages-tail train. The height and width of the model are set in proportion to the actual EMU. According to the experience of dealing with related problems, the simplified model has little change in the basic distribution law of the flow field compared with the complete EMU model.
(2) Due to the complex structures of pantographs, door handles, bogies, etc., the structures that affect the grid division are ignored as much as possible, and smooth surfaces are simplified to replace these structures when processing.

**Figure 1:**
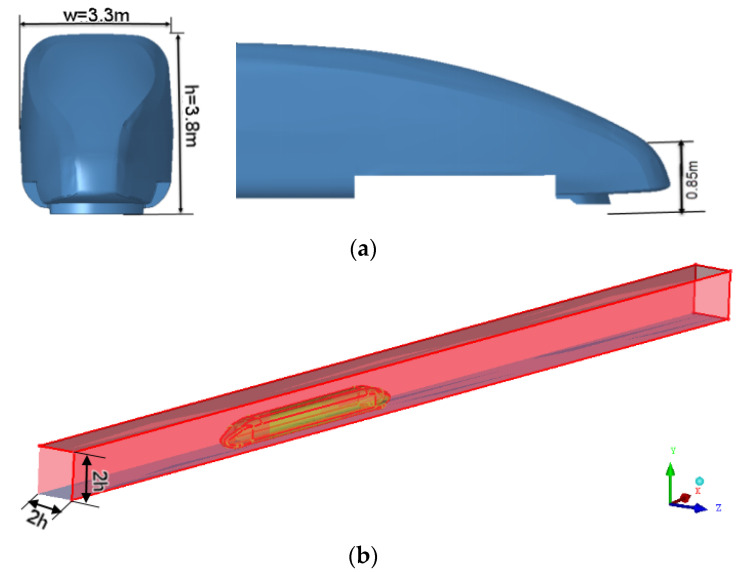
Streamlined locomotive and computational domain. (**a**) Streamlined locomotive; (**b**) computational domain.

Different from the semicircular tunnel used in other documents, the external flow field of the cuboid is directly selected as the calculation domain. It can be compared with the existing literature: whether different tunnel models will have different effects on the tail flow of multiple units. Considering that the front of the vehicle should be a certain distance from the entrance boundary, the proportional coefficients in the three directions are set to 4, 2, and 2, respectively, which simulate the operation of the EMU in the vacuum pipeline. Set the entrance of the flow domain as the velocity inlet and the speed as 800 km/h; then, the corresponding Mach Number is greater than 0.3. The air should be regarded as a compressible fluid. The outlet of the flow domain is set as a pressure outlet, and the relative pressure is 0. This article discusses the operation in the vacuum pipeline, and the operating pressure is set to 0.01 atm, which is 1000 Pa. In order to facilitate the calculation and reduce the occupation of computer resources, the surface of the EMU model, the vacuum pipeline, and the ground are set as fixed walls, and the calculation domain is shown in Figure 1.

### 2.3. Meshing

**Figure 2:**
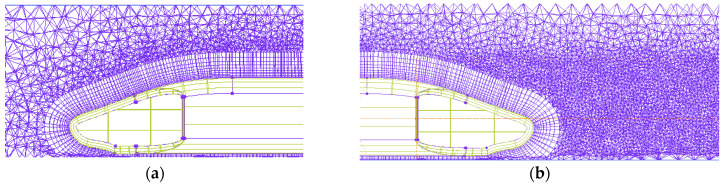
Schematic diagram of the local grid: (**a**) train head; (**b**) train tail.

**Figure 3:**
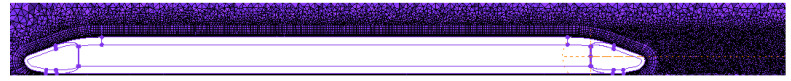
Schematic diagram of the grid around the car body.

**Figure 4:**
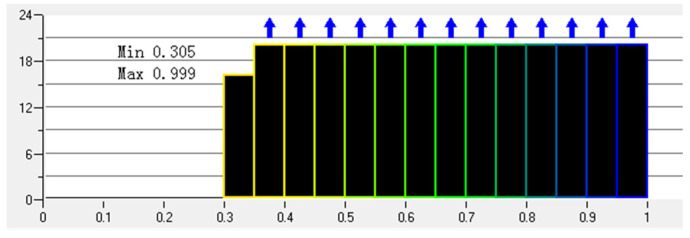
Mesh quality.

In order to simulate and obtain as much as possible the air flow outside the EMU model and the air flow near the wake area, the boundary layer grid of the EMU model car body and the grid near the wake area need to be encrypted. The settings are as follows:

(1) Set the global grid size and scale factor.
(2) Set the prism for the car body. The boundary layer grid is a triangular prism grid. Set the height, height ratio, and number of layers. A schematic diagram of the local grid of the front of the car is shown in Figure 2.
(3) Set the grid size of the encryption zone. The schematic diagram of the grid around the car body is shown in Figure 3. The minimum mesh quality is 0.305, which is an ideal mesh quality. The mesh quality is shown in Figure 4.

### 2.4. Simulation Method

In this paper, the area of the vacuum tube high-speed train wake flow field is carefully explored, that is, it is necessary to capture sufficient flow information around the high-speed train flow field. Considering the computing power and efficiency of the computer, the IDDES simulation method is used to simulate the turbulent flow of the model. The IDDES model is developed from the DES model, which can realize the transformation of the processing grid between LES and RANS during model solving and simulation. Its expression is as follows:

(3) $$L_{DES}={\tilde{f}}_{d}(1+f_{e})L_{t}+(1-{\tilde{f}}_{d})C_{DES}L_{g}$$

(4) $${\tilde{f}}_{d}=\max((1-f_{d}),f_{B})$$

where ${\tilde{f}}_{d}$ is the conversion function; $f_{d}$ is the conversion function of the DDES model; $f_{B}$ is the conversion function of wall modeled LES (WMLES);

$f_{e}$ is the control function of wall simulation as follows:

(5) $$f_{e}=\max((f_{e1}-1),0)f_{e2}$$

During grid size calculation, the influence of wall distance was taken into account:

(6) $$L_{g}=\min(\max(C_{w}d_{w},C_{w}h_{\max},h_{wn}),h_{\max})$$

where $h_{wn}$ is the step length of grids; $h_{\max}=\max(\Delta x,\Delta y,\Delta z)$; $C_{w}$ is a coefficient, and normally set to 0.15 in the IDDES model.

In addition, the flow field is numerically solved by an implicit coupling solver. The convection term adopts the second-order upwind scheme; the second-order implicit scheme is used for time discretization accuracy; Gauss’ least squares method is used for calculation.

### 2.5. Reliability Analysis

**Figure 5:**
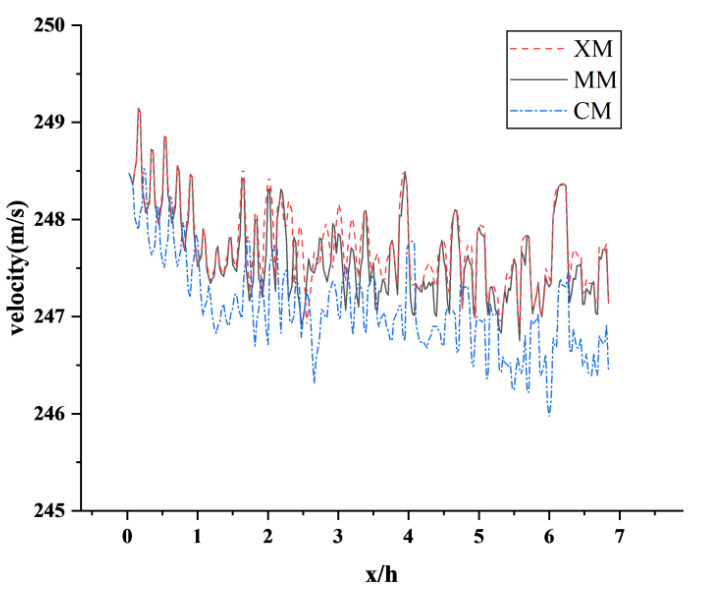
Curves of velocity along the flow direction in the near wake region of the three schemes.

**Figure 6:**
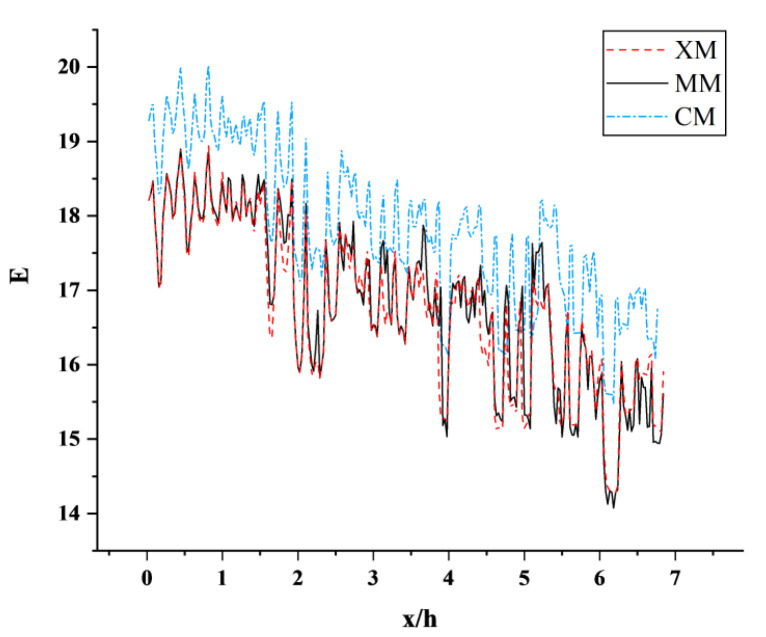
Variation curve of turbulent kinetic energy along the flow direction in the near-wake region of the three schemes.

The total number of three grid models of atm1 is 3.2 × 10^6^, 5.8 × 10^6^ and 8.3 × 10^6^, as shown in Table 1. The coarse grid, medium grid, and fine grid (CM, MM, XM) of atm1 are selected for grid independence verification. According to the position of the model in the calculation domain, the flow direction position and transverse position of the tail car nose are set as x = 0 and z = 0. Figure 5 shows the speed of the high-speed train near the wake area along the flow direction. The results of the monitoring points in the wake area of the high-speed train of the three schemes show that XM and MM are in good agreement, the change trend is basically the same within the flow direction distance, and the speed trend gradually decreases. Although the deviation occurs at 3 h, the overall error is small. The change trend of CM and the former two are roughly similar, but the curve deviates significantly. Figure 6 shows the variation of turbulent kinetic energy in the near wake region of a high-speed train along the flow direction. The three schemes can obviously capture the turbulence information in the wake flow field of a high-speed train. Among them, the simulation results of monitoring points of MM and XM schemes are similar. Considering the comprehensive calculation efficiency, the MM grid encryption scheme is selected as the simulation calculation model of the vacuum pipeline working condition of a high-speed train.

In order to verify the accuracy of the numerical method and the car body model, the MM scheme calculation model is used to simulate the aerodynamic drag characteristics and other related parameters of the high-speed train running in the vacuum pipeline. The pressure in the vacuum pipeline is set to 0.01 atm, the speed of the EMU is 600 km/h, the ambient temperature is 300 K, and the remaining boundary conditions are consistent with the above. The drag coefficient of the EMU is expressed by the dimensionless coefficient as follows:

(7) $$C_{d}=\frac{F_{d}}{0.5\rho U^{2}S}$$

where *F_d_* is the drag of the EMU in operation, *ρ* is air density, *U* is the speed of the EMU, and *S* is the cross-sectional area of the EMU.

**Figure 7:**
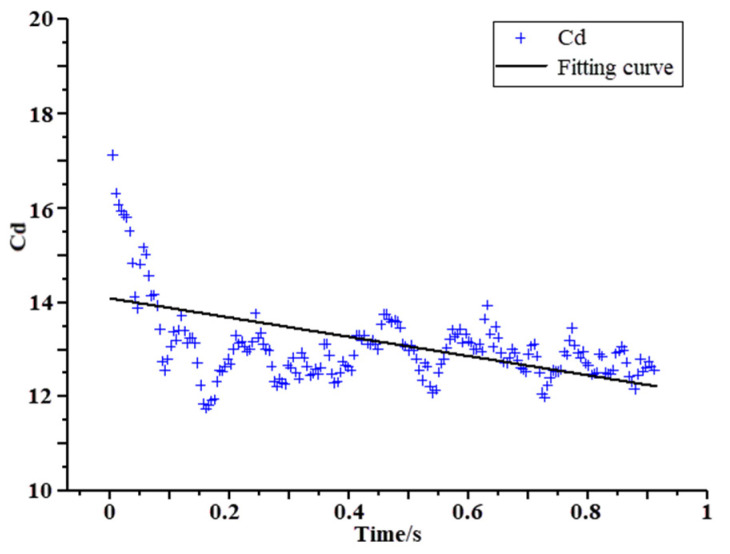
Aerodynamic drag coefficient *C_d_* scatter and fitting curve in 1 s.

Shown in Figure 7 is the scatter diagram of the aerodynamic drag coefficient *C_d_* of the vacuum tube EMU in 1 s. The *C_d_* coefficient is mostly distributed near a straight line with an intercept of 14.079 and a slope of −2.039. More than 80% of the *C_d_* is between 12.1 and 13.7. The maximum error of the *C_d_* value during the operation of the vacuum tube in the literature [79] is within 8%, indicating that the algorithm and model in this paper are correct and reliable and can meet the calculation requirements of the turbulence characteristics in the wake region.

## 3. Turbulence Characteristics Analysis

### 3.1. Wake Vortex Structure

The Q-criterion is a classical criterion based on the Galilean invariant vortex definition. With the lowest pressure as the additional condition, the vortex is defined by the positive value of the second invariant *Q* of the velocity gradient. The theoretical expression is:

(8) $$Q=-\frac{1}{2}({\left(\frac{\partial u}{\partial x}\right)}^{2}+{\left(\frac{\partial v}{\partial y}\right)}^{2}+{\left(\frac{\partial w}{\partial z}\right)}^{2})-\frac{\partial u}{\partial y}\frac{\partial v}{\partial x}-\frac{\partial u}{\partial z}\frac{\partial w}{\partial x}-\frac{\partial v}{\partial z}\frac{\partial w}{\partial y}$$

**Figure 8:**
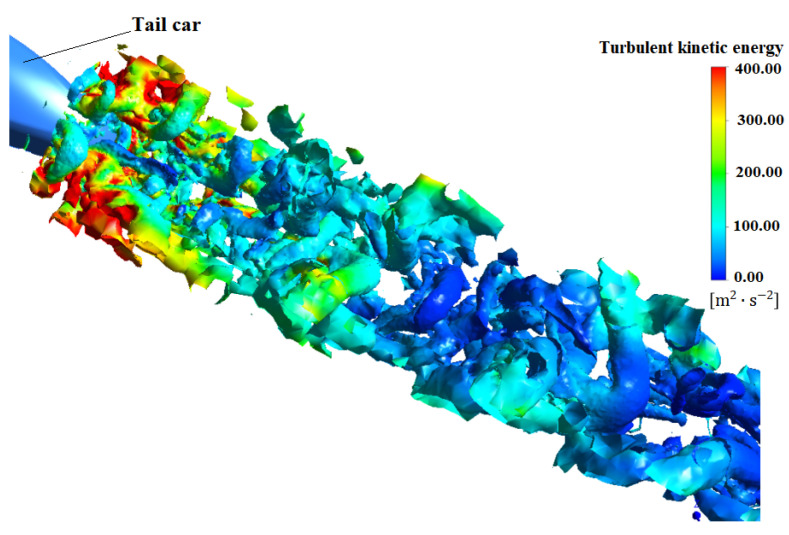
Vortex distribution in wake region of atm1 at 800 km/h.

It can be seen from Figure 8 that this pair of vortices is near the symmetrical position z = 0 near the wake area of the rear of the vehicle at 800 km/h. With the extension of the longitudinal (x direction), this pair of vortices moves horizontally to both sides. An obvious negative pressure zone appears at the vortex core.

Moreover, Figure 8 is the wake vortex of atm1 model of high-speed train obtained by using Q-criterion and velocity as characteristic scale. According to the location of the train model, the flow position and transverse position of the tail car nose are set to x = 0 and z = 0. From the figure, it can be seen that there is a strong vortex in the near wake region of the tail of the car, and it continues to fall off from the tail end. In the process of downstream propagation, it presents a symmetrical distribution, in which the vortex away from the tail car is gradually increasing, but from the characterization of speed, the strength of the vortex is gradually decreasing.

### 3.2. Train Wind

**Figure 9:**
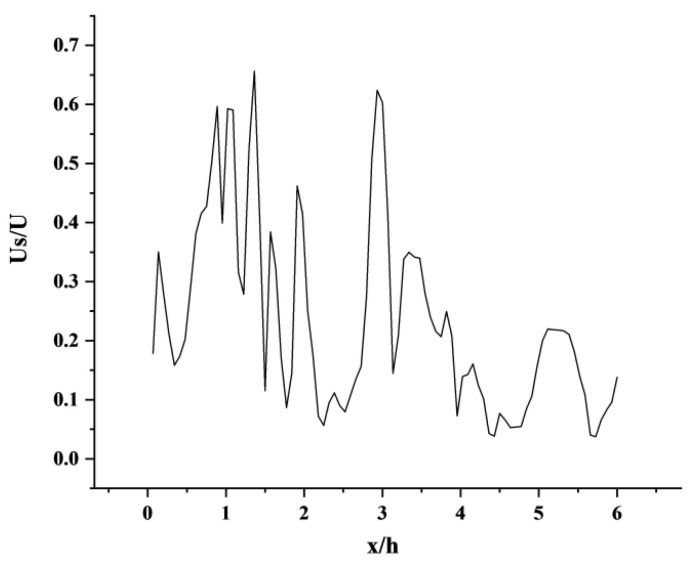
Train wind speed along the flow direction in the wake area.

The development of train wind is closely related to the vortex structure. Since the EMU model used in this article is stationary, the train wind speed is defined as follows:

(9) $$u_{s}=\sqrt{{\left(U_{\infty }-u\right)}^{2}+w^{2}}$$

where u is the instantaneous flow velocity, ω is the instantaneous flow velocity in the span direction. Figure 9 and Table 2 show the comparison between the numerical simulation results and the experimental train wind speed under atm1 conditions, and the speed is dimensionless.

It can be seen from Figure 9 that when the train is in the vacuum pipeline, it will be affected by the tube wall, the speed curve is constantly oscillating, and the velocity peak appears at the flow distance of 1 h, 3 h, and 5 h. Comparing the literature [80], we can see that the two have similar regularity. The simulation data of the open line shows that at x = 0, that is, a small oscillation peak appears in the wind speed of the train at the nose of the trailing car, and the speed peak appears again around the downstream position of x = 5 h. In this paper, the dimensionless train wind speed value is higher than the speed value in the literature [80]. The vortex away from the chaser is gradually increasing, but from the speed characterization point of view, the strength of the vortex is gradually decreasing.

### 3.3. Analysis of Aerodynamic Characteristics

#### 3.3.1. Aerodynamic Characteristics of atm1

**Figure 10:**
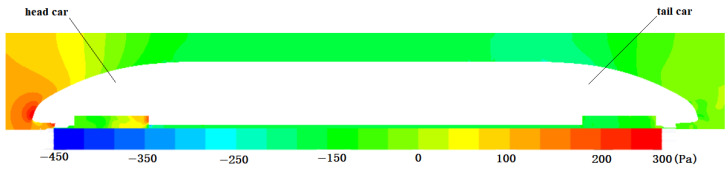
Pressure field distribution of EMU head and tail.

Figure 10 shows the pressure field of the front and rear of the EMU model at 800 km/h and 0.01 atm. It can be seen from the figure that there is a maximum pressure near the nose tip, and the pressure gradually decreases backward along the body. Negative pressure appears at the intersection of the front and the body, indicating that vortex shedding occurs here. The pressure distribution of the tail car is similar to that of the head car. The pressure gradually decreases along the car body, and negative pressure appears in the transition area where the uniform section body and the variable section tail intersect.

**Figure 11:**
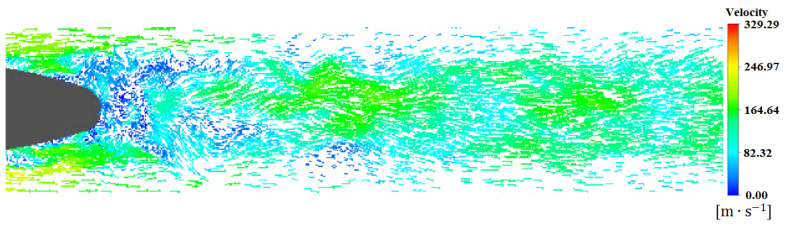
Speed vector at the rear space of the tail car.

**Figure 12:**
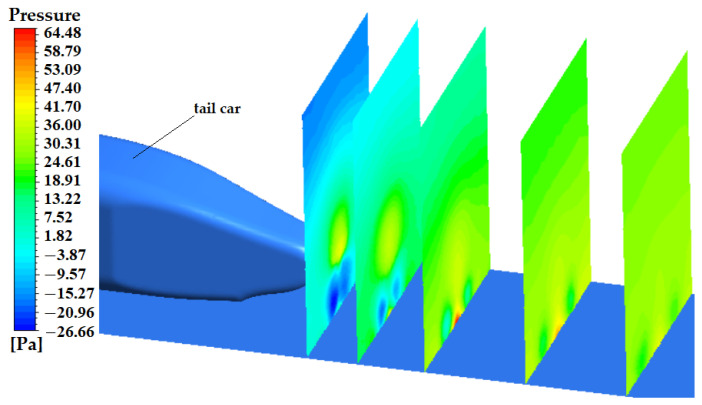
Sectional pressure in the wake region of the tail.

Figure 11 shows the local velocity vector at the rear space of the tail car. It can be seen when the train is running at high speed, because the air is viscous, the air at the rear of the train will move with the train, thus forming a wake area, which is far affected along the flow direction. In Figure 12, five sections are cut perpendicular to the ground in the wake area of the train to show the pressure distribution at the section. These five sections are arranged in the wake area according to a certain growth ratio. The effect of pressure fluctuations of the vortex on the ground can be observed. During the downstream propagation of the streamwise vortex, due to the interaction between the ground effect and the vortex, it will move vertically downward and simultaneously horizontally outward, approximately symmetrically distributed, and exhibit periodic shedding similar to the Karman vortex street.

#### 3.3.2. Drag Analysis of Three Different Length Models

Table 3 shows the aerodynamic drag of three train models with different marshalling lengths when the pressure of the pipeline and the speed of the train are constant. Because the train model is simplified, the calculated resistance value will be smaller than the real value, but the obtained law is accurate. With the increase of the characteristic length of the EMU model in a vacuum pipeline, the aerodynamic drag, including the pressure drag and viscous drag, increases. Compared with atm1, the aerodynamic drag, pressure drag, and friction drag of atm2 increase by 67.71%, 65.22%, and 73.08%, respectively. Compared with atm2, the aerodynamic drag, pressure drag, and friction drag of atm3 increased by 17.91%, 13.75%, and 26.48%. This shows that on the basis of long-grouped vacuum pipeline trains, when the same length of carriages is added, the increment of aerodynamic resistance is smaller than that of short-grouped trains. Additionally, this increment will decrease with the increase in train length.

**Figure 13:**
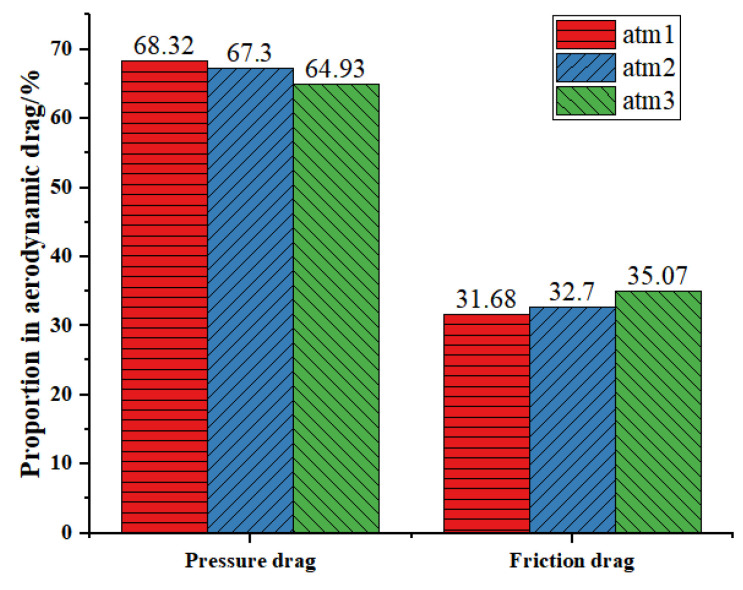
The proportion of pressure and friction drag in aerodynamic drag.

Figure 13 shows the proportion of pressure drag and friction drag in aerodynamic drag of three different train models. Overall, the pressure drag of the three models accounts for 60~70% of the aerodynamic drag, and the friction drag accounts for 30~40% of the aerodynamic drag. When the length of the train model increases, the surface area of the train body also increases, so the proportion of friction drag becomes larger, and the contribution of friction drag to aerodynamic drag is gradually increasing.

### 3.4. Analysis of Turbulence Kinetic Energy Analysis

#### 3.4.1. Under atm1 Conditions

**Figure 14:**
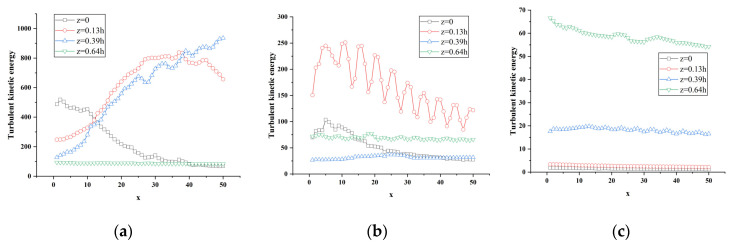
Variation of turbulent kinetic energy along the flow direction at different spanwise positions of atm1. (**a**) y = 0.18 h; (**b**) y = 0.36 h; (**c**) y = 0.5 h.

When the vertical position y is 0.18 h, 0.36 h, and 0.5 h, and z is at the positions of 0, 0.13 h, 0.39 h and 0.64 h, respectively, the turbulent kinetic energy along the flow direction changes curve as shown in Figure 14. In Figure 14a, the flow direction range 0 < x < h and the vertical position y = 0.18 h, the spanwise position z = 0 shows an overall decreasing trend in which the curve rises first and then drops after a peak at x = 2; the position of z = 0.64 h is relatively flat, but it can be seen from the partial enlarged view of Figure 13a that the curve first drops and then rises, and then drops and rises after reaching the peak repeatedly. The overall trend shows a decreasing trend at the same position as z = 0. The curve of z = 0.13 h gradually rises first and starts to rise gently at x = 25, and the turbulent kinetic energy gradually decays after reaching a peak of about 840 J; the curve of z = 0.39 h shows an upward trend and continues to fluctuate and rise after reaching a peak. Among them, the turbulent kinetic energy at z = 0 before x = 10 is more active than the other three, and the turbulent kinetic energy at z = 0.13 h between 10 < x < 40 is more active than the other three. However, the turbulent kinetic energy curve of z = 0.39 h is much higher than the other three at x = 40.

Figure 14b shows the turbulent kinetic energy flow direction change curve at different spanwise positions when the vertical position is at y = 0.36 h. It can be clearly seen from the figure that there is an obvious regular change in the curve at the spanwise position z = 0.13 h: after the curve rises abruptly and peaks, it gradually drops, repeating in sequence, but it can be seen that the peak is constantly decreasing, and the curve as a whole shows a decline. The trend indicates that the turbulent kinetic energy is considerable, and the turbulent kinetic energy is continuously dissipated and decayed during the development of the flow direction; the curve z = 0 is similar to the curve z = 0.13 h. After a certain distance along the flow direction, the turbulent kinetic energy becomes active, and the curve gradually rises and begins to attenuate after the peak appears. In combination with Figure 14b, it can be seen that the curve at the spanwise position z = 0.64 h also has a similar rule to the former; the curve at the spanwise position z = 0.39 h gradually rises, reaching about x = 20 It decays after the peak, and starts to rise steadily after x = 25.

Figure 14c shows the change curve of the turbulent kinetic energy of the flow at different spanwise positions at the vertical position y = 0.5 h. It can be seen from Figure 8d that the turbulent kinetic energy at positions z = 0 and z = 0.13 h still has the above-mentioned regular pattern at this time. In the spanwise position z = 0.39 h, the curve fluctuation rises and reaches the peak at x = 13 (x = 0.256 h), and then the fluctuation drops; the turbulent kinetic energy at z = 0.64 h is much higher than the other three, and the maximum turbulent kinetic energy can reach 66 J. From Figure 13c, it can be found that the turbulent kinetic energy at the four spanwise positions at the vertical direction y = 0.5 h is constantly attenuating the distance along the flow direction. When the vertical position is higher, the turbulent kinetic energy is mainly concentrated on both sides of the middle section, that is, the position where the absolute value of z in the span direction is larger.

**Figure 15:**
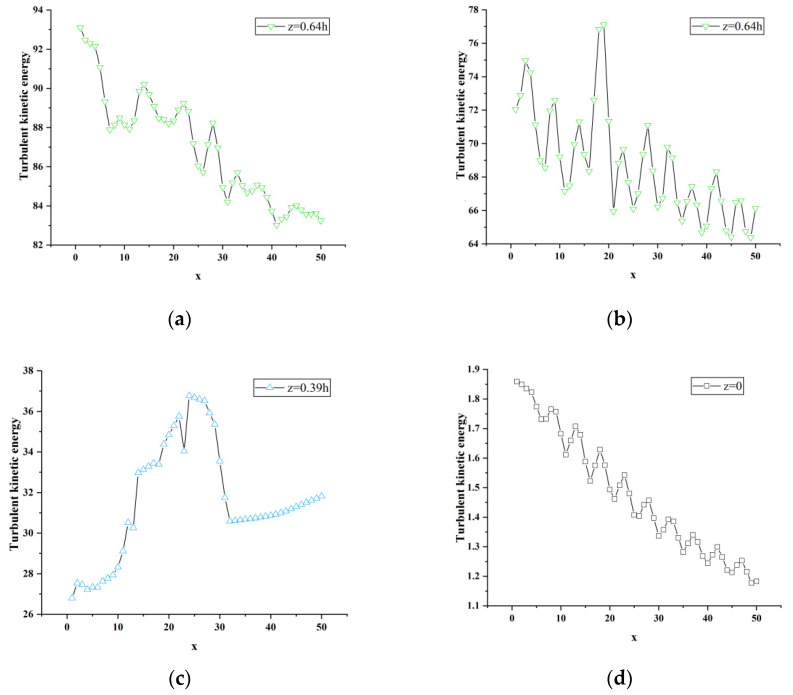
Local enlargement of atm1 turbulent kinetic energy curve. (**a**) y = 0.18 h, z = 0.64 h; (**b**) y = 0.36 h, z = 0.64 h; (**c**) y = 0.36 h, z = 0.39 h; (**d**) y = 0.5 h, z = 0.

A longitudinal comparison of the turbulent kinetic energy curves at three different vertical positions in Figure 15 shows that each vertical position has the maximum value of turbulent kinetic energy (y = 0.18 h is about 935 J; y = 0.36 h is about 248 J; y = 0.5 h is about 67 J) is gradually decreasing, and the energy of the wake vortex is constantly attenuating, indicating that the wake vortex in the near-wake region is mainly concentrated near the lower end of the nose tip near the ground and continues to propagate downstream.

#### 3.4.2. Under atm2 and atm3 Conditions

**Figure 16:**
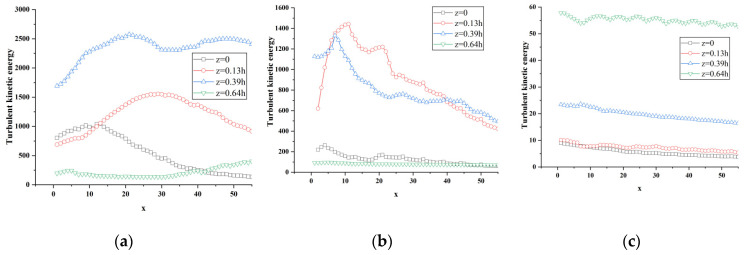
Variation of turbulent kinetic energy along the flow direction at different spanwise positions of atm2. (**a**) y = 0.18 h; (**b**) y = 0.36 h; (**c**) y = 0.5 h.

**Figure 17:**
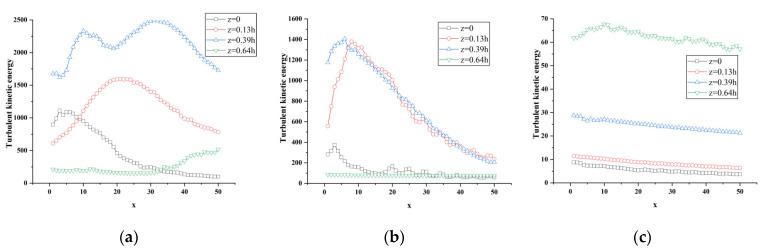
Variation of turbulent kinetic energy along the flow direction at different spanwise positions of atm3. (**a**) y = 0.18 h; (**b**) y = 0.36 h; (**c**) y = 0.5 h.

**Figure 18:**
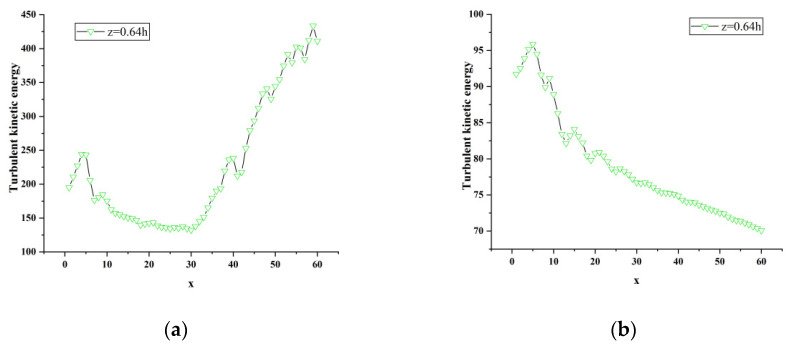
Partial enlargement of atm2 turbulent kinetic energy curve. (**a**) y = 0.18 h, z = 0.64 h; (**b**) y = 0.36 h, z = 0.64 h.

**Figure 19:**
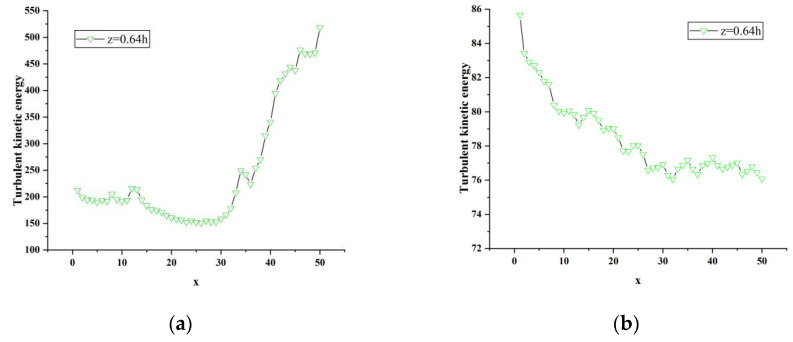
Partial enlarged view of atm3 turbulent kinetic energy curve. (**a**) y = 0.18 h, z = 0.64 h; (**b**) y = 0.36 h, z = 0.64 h.

Figure 16, Figure 17, Figure 18 and Figure 19 show the change curve and partial enlarged view of the turbulent kinetic energy along the flow direction under atm2 and atm3 conditions. Comparing the changes of the turbulent kinetic energy flow in the wake area under three different characteristic lengths, it can be found that the turbulent kinetic energy curve at each position is similar to the above-mentioned change trend, and the fluctuations begin to attenuate or increase after reaching their respective peaks.

## 4. Conclusions

In this paper, the IDDES method is used to simulate the flow field around the simplified model of high-speed EMU based on vacuum pipes, so as to obtain and analyze the aerodynamic resistance of high-speed EMU and its turbulent characteristics in the near wake region. The relevant conclusions are as follows:

(1) There are strong vortices in the wake area, and the energy carried by them is concentrated at the lower vertical position. The tail vortex is falling off from the tail end. In the process of propagating downstream, the vortex structure away from the tail vehicle is gradually increasing, but from the characterization of velocity, the strength of vortex is gradually decreasing.
(2) When the vertical position is closer to the ground, the train wind shows a chaotic state, and the vortex has a large energy concentration near the ground. In the wake area, the train wind speed peak appears many times, but the peak value is gradually decreasing, and the overall trend is gradually decreasing.
(3) When the train speed is constant, with the increase of the characteristic length of the EMU model, the train surface area increases, the proportion of differential pressure resistance decreases gradually, the proportion of viscous resistance in aerodynamic resistance is more significant, and the contribution to aerodynamic resistance increases.
(4) The turbulent kinetic energy of the three train models with different lengths is basically the same, gradually decreasing and then rising to the peak, repeating many times and gradually decreasing. The maximum turbulent kinetic energy of each vertical position is also gradually declining, indicating that the wake vortex in the near wake area is mainly concentrated near the lower end of the nose tip near the ground, and continues to propagate downstream and move horizontally.

## Figures and Tables

**Table 1:** Maximum size of grid encryption unit in wake area.

Maximum Size of Grid Cell	CM	MM	XM
Region around body	0.32 h	0.02 h	0.015 h
Region of tail flow field	0.105 h	0.08 h	0.04 h
Total number of grids (10^6^)	3.2	5.8	8.3

**Table 2:** Comparison of numerical simulation and peak wind speed of test train.

Method	X	Y	Z	Peak Train Wind Speed
IDDES	5 × 10^5^	0.18	0.3	0.66
Test (track side)	6 × 10^5^	0.05	2.0	0.23
Test (station)	6 × 10^5^	0.35	2.0	0.15
LES	3 × 10^5^	0.05	2.0	0.31

Note: The experimental data and LES data come from literature [80]. Where Y represents the vertical position, the test data is based on the rail surface, and this article is based on the ground of the vacuum pipeline; Z represents the ratio of the spanwise position to the half-width w/2 of the model.

**Table 3:** Drag comparison of atm1, atm2, and atm3.

Model	Pressure Drag (N)	Friction Drag (N)	Aerodynamic Drag (N)
atm1	1207.24	559.88	1767.12
atm2	1994.65	969.03	2963.68
atm3	2268.87	1225.62	3494.49

## Data Availability

Not applicable.
